# Science and Society: Pathways to Equitable Access and Delivery of Genomics Medicine in Africa

**DOI:** 10.1007/s40142-024-00211-0

**Published:** 2025-02-24

**Authors:** Nchangwi Syntia Munung

**Affiliations:** Division of Human Genetics, Faculty of Health Sciences, University of Cape Town, Cape Town, South Africa

**Keywords:** Genomics for All, Genomic Medicine, Science diplomacy, Ethics and Equity, Science Advocacy, Africa

## Abstract

**Purpose of Review:**

Recent advances in genetics are pushing the frontiers of health research in Africa. Notable developments include the release of the draft human pangenome reference, regulatory approval of gene editing therapies for sickle cell disease, and the announcements of major initiatives such as the Ghana Genome Project, the Personalized Medicine in North Africa Initiative, Nigeria’s 100K Genome Project and South Africa’s 110K Human Genomes Project. Additionally, gene-based therapies for HIV are on the horizon, with clinical trials planned in some African countries. Despite this progress, a pressing challenge remains: ensuring equitable access and delivery of genomics medicine worldwide, particularly in Africa and other low and middle income regions.

**Summary and a Call to Action:**

Science diplomacy and academic-industry partnerships are key to achieving "Genomics for All." This requires collaboration between African governments, academic institutions, funding agencies, commercial biotechnology companies, civil society, and international health organizations. Together, these stakeholders must define and establish a sustainable framework to support genetic research in Africa, increase the availability of genetic data from African populations, and set-up translational genomics medicine initiatives tailored to the continent’s unique healthcare needs. Science advocacy and diplomacy is also needed to establish mechanisms that prevent the hoarding of genetic resources, including genetic data and novel interventions, and guarantee equitable access to the scientific, medical and economic benefits of genomics for all nations. Achieving this vision may necessitate international treaties to promote equitable access to genomic innovations, responsible and ethical cross-border data sharing, and long-term strategies to address funding gaps in genomic research and its application in medicine and healthcare in Africa.

## Introduction

The field of genomics has undergone remarkable advancements over recent decades, marked by milestones such as the launch of Human Genome Project in the 1990s [1] and the publication of the first reference human genome in 2002 [2]. Recent scientific developments that have significantly expand the frontiers of human genetics include the release of a draft human pangenome reference [3]; regulatory approval of gene editing therapies to cure sickle cell disease (SCD), beta thalassemia, haemophilia, and Duchenne muscular dystrophy [4–6] and the announcement of the South African 110k Human Genomes Project, the Ghana Genome Project and the Nigerian 100K genome project [7]. Additionally, gene-based therapies for HIV are on the horizon, with clinical trials planned in select African countries [8]. These developments signal an exciting era in genomic research and precision medicine. However, a pressing challenge remains: how can we ensure that the benefits of genomics are equitably distributed worldwide, particularly in regions like Africa with limited resources to support scientific research and innovation? That is, how can we advance the vision of "Genomics for All." For example, gene therapy trials for SCD and haemophilia have shown promising results [9–11], yet most SCD patients in Africa are unlikely to access these, despite the continent bearing a disproportionate share of the global SCD burden.

“Genomics for All” [12] is a vision aimed at ensuring that genomic technologies and their benefits are accessible to all people and nations, regardless of their geographical location, economic status, scientific standing or biotechnological and health infrastructure. Achieving this goal requires a multifarious strategy encompassing: international cooperation to support research exploring the ancestral genetic diversity of global populations; responsible sharing and access to genomic data; developing affordable genomic technologies; and equitable access to genomic medicine by peoples of all nations. It is also about developing policies that promote responsible genomic research and innovation and ensures that the benefits of genomic advancements are shared equitably across nations.

Science diplomacy, particularly diplomacy for science, which leverages scientific collaboration between nations and stakeholders to solve societal challenges, offers a promising pathway to achieve the vision of “Genomics for All.” By leveraging collaboration between nations and stakeholders, including national governments, academic institutions, scientific and healthcare industries, international health agencies, philanthropists, and research funders, science diplomacy can help build sustainable genomics medicine initiatives in Africa.This paper presents recommendations and initiatives necessary to realise the vision of “Genomics for All,” with a focus on leveraging science diplomacy to enable access to genomic healthcare solutions for African populations.

## The Diversity Imperative: Addressing Gaps in Availability of Genetic Data from Different Populations

Despite significant advances in genomic research, concerns persist about its potential to exacerbate global health and biotechnology inequities [13, 14]. One major issue is the underrepresentation of certain population groups in genomic studies. This lack of representation risks perpetuating inequities in genomic medicine, as many genetic therapies and interventions are developed using data primarily derived from populations of European descent, thereby limiting their efficacy for other racial and ethnic groups. Moreover, the limited availability of allele frequency databases for underrepresented populations hinders variant interpretation, exacerbates diagnostic disparities, and impedes efforts to improve clinical outcomes for patients from some population groups [15].

Addressing the diversity imbalance in genomics requires a coordinated global strategy to generate genetic data for underrepresented populations, particularly those of African ancestry and indigenous groups. Initiatives such as the African Genome Variation Project, H3Africa [16], the 100 K Nigerian genome project [7], the Gambian genome project and the Southern African Human Genome program [17] have been instrumental in generating genomic data on African populations. Other planned efforts include the Ghana Genome Project, the Genome Tunisia project [18], the vision to sequence three million genomes across Africa [19]; the African pharmacogenomics consortium [20] and the anticipated launch of the South African 110K genome project, which plans to include 10,000 participants living with rare diseases. These initiatives have been made possible through various forms of international cooperation, demonstrating how science diplomacy can help advance the genomics agenda in Africa. For instance, H3Africa was the brainchild of a collaboration between the African Society of Human Genetics, African diaspora scientists, the US National Institutes of Health, the Wellcome Trust in the UK, the African Academy of Sciences, and several African research institutions and universities. Similarly, the Personalized Medicine in North Africa (PerMediNA) initiative is a partnership between Northern Africa scientists/academic institutions and the French Ministry for Europe and Foreign Affairs [21]. The Nigerian 100K genomes project, on the other hand is a partnership between the Nigerian-based genomics startup, 54gene and the African Center for Translational Genomics, a nonprofit organisation and exemplifies the importance of academic-industry partnerships in science.

## Minimizing Data Hoarding: Adopting a Culture of Maximum Genomic Data Sharing

Data sharing is crucial for advancing genomics research and innovation. While a number of international genomics initiatives in Africa support data sharing and have data sharing policies, the effectiveness of these policies in achieving maximum data sharing and access remains unknown. For example, the H3Africa data sharing policy seeks to enable fair data sharing while minimising the exploitation of African scientists [22]. The policy includes an eleven-month publication embargo, allowing H3Africa investigators to analyse and share data internally, before the genomic and phenotypic data are made available to the broader scientific community under controlled access. However, such embargoes and restrictive access protocols, though designed to protect African researchers, may inadvertently encourage data hoarding. Moreover, a recent study revealed that many biomedical researchers who publicly committed to data sharing failed to provide access when contacted [23].

A further challenge to genetic data sharing relates to compliance with personal data protection laws. Many African countries have enacted legislation that classifies genetic data as sensitive, requiring stringent safeguards for processing [24]. While these legislations are key to safeguarding the privacy of data subjects, overly restrictive data protection laws can stifle global health research [25]. Furthermore, data-sharing discussions in international projects often focus on transferring African-generated data to Global North institutions. This raises the following critical questions: Are genetic datasets from the Global North relevant to African research priorities? Or do restrictive policies in the Global North limit data sharing with African scientists? To foster a culture of maximum data sharing globally, African genomics initiatives must revisit current data sharing practices and policies address barriers to data sharing, and identify and implement incentives for data sharing. Alignment with open data-sharing practices should be encouraged, however, open data sharing policies should be paired with data stewardship models that empower African researchers to maintain control over their research data. Innovative technical approaches such as data federation and data visiting [26] that allow for shared data analysis without compromising local ownership and control of data should be prioritised. Additionally Regional data sharing frameworks like those proposed by the Economic Community of West African States (ECOWAS) and the Southern African Development Community (SADC) provide a foundation for advocating for international agreements that promote the responsible sharing of human genetic data for research and innovation.

## Giving Voice to Data Subjects: Public Engagement and Education in Genomics

Data subjects, that is patients, research participants and study communities are often overlooked in discussions about genetic data governance. While health research ethics committees typically oversee consent for the secondary use of research data, on behalf of data subjects, it is critical to recognise the need for continuous engagement with data subjects regarding how their data is used. Many African research participants are open to data sharing, but establishing mechanisms for ongoing engagement ensures that data subjects can modify their consent preferences as their circumstances or willingness to participate in research evolves. Dynamic consent [27] and public education in genetics offer promising solutions as they provide data subjects with greater control over their data. It keeps the data subject informed about new studies using their data and enables them to adjust their sharing preferences over time. Such practices not only uphold individual autonomy but also builds trust between researchers and the public. Similarly, public education in genetics and secondary data use for health research not only empowers data subjects to make informed decisions about the use of their data but provides an avenue for giving voice to communities that have voluntarily contributed their genetic information for the benefit of science and medicine.

Patient and public engagement are also essential to the success of genomics medicine initiatives [28, 29]. Public engagement programs should allow for the incorporation of diverse perspectives, including indigenous knowledge about genetic diseases, into the research design and implementation. It should also empower communities with a foundational understanding of genetic concepts, the implications of sharing genomic information, the potential applications of genomics in healthcare, and the ethical and social issues in genomics [30, 31]. 

Efforts to improve genomic literacy in Africa have gained momentum, driven by initiatives such as Genome Adventures program [32] by H3Africa’s Collaborative African Genomics Network [33], which uses animated storytelling to improve public understanding of genomics. Similarly, many research projects now embed community engagement activities into their design to build trust and get buy-in during project implementation. However, more inclusive participation is now evident in recent initiatives, such as a public engagement project on heritable human genome editing in South Africa [34] and the DS-I Africa funded project on public engagement for genomics and big data in Medicine (PUBGEM-Africa) [35]. Lessons can also be drawn from other initiatives outside Africa, such as the Genomics England participant panel which integrates the perspectives of research participants into genetic medicine services and research [36]. 

Beyond education and engagement, science advocacy efforts are also needed to address the affordability and accessibility of genomic solutions for medicine and healthcare in Africa, ensuring that advancements in genomics benefit all populations equitably.

## Enabling Translational Genomics Medicine Research Initiatives

The public health impact of genomics research projects in Africa will ultimately depend on their ability to translate research findings into equitable access to genomic medicine across the continent. Presently, basic genetic medicine services, such as genetic screening and counseling for conditions like SCD, are largely unavailable in many African countries, even within tertiary healthcare facilities. Similarly, while groundbreaking advancements like gene therapy may offer life-changing outcomes for individuals living with SCD, [9, 11], the prohibitive cost (approximately $2 million per patient) and the near-total absence of infrastructure to conduct SCD gene therapy trials in most African countries [37, 38], render these treatments inaccessible to the vast majority of patients in Africa.

 Investment in translational genomics research in Africa can accelerate the development of locally relevant, cost-effective genetic medicine services tailored to the continent’s public health needs. While some genomics research projects have made notable strides by integrating genetic medicine services into their research projects, much more work is required to convert research outcomes into actionable, affordable and accessible healthcare solutions for African communities. The World Health Organization's recent report on *accelerating access to genomics for global health* [39] provides a useful framework for bridging this gap. The report acknowledges the stark disparities in widespread availability of genomic technologies in wealthier nations and their scarcity in resource-limited settings. It also outlines a solution centered on international cooperation and partnerships among non-governmental organizations, academic societies, WHO member states, academic institutions, and biotechnology companies, to drive access to genomic medicine services for people living in low resource settings.

## Development of Genomic Medicine Training and Clinical Programs in Africa

Establishing genomic medicine programs in Africa necessitates collaborative, multidisciplinary efforts involving biomedical scientists, medical geneticists, data scientists, genetic counselors and bioethicists. However, many African healthcare systems lack an interdisciplinary workforce to man genetic medicine services. Nontheless, encouraging progress is being made in building Africa’s genomic medicine workforce. For example, the University of Witwatersrand, South Africa offers a one-year, full-time Master of Science in Genomic Medicine at the University of Witwatersrand; and the African Genomic Medicine Initiative has introduced blended-learning short courses in genomic medicine for nurses and pathologist [33]. A couple of universities in South Africa and Ghana now offer postgraduate programs in genetic counseling, and H3Africa has trained an appreciable number of African scientists and clinicians across disciplines such as human genetics, data science, genetic counseling, bioinformatics and bioethics.

Genomic education for healthcare providers is a global priority, and African countries must tailor training programs to their specific health systems and needs. A survey by the Global Genomic Medicine Consortium [40] identified training medical geneticists, genetic counselors, and laboratory scientists as a priority for advancing the genetic medicine agenda in LMICs. The survey also highlighted the necessity of revising medical curricula to integrate genetics education. Given the pivotal role of primary healthcare in many African countries, platforms for the continuous education of primary healthcare providers in the scientific, clinical, and ethical aspects of genetic medicine will also be critical.

A strategic and cost-effective way to advance clinical genomic medicine programs in Africa is by designing research initiatives that balance short-term clinical utility with long-term research outcomes. This would allow for the timely integration of genetic medicine into the existing healthcare programs. Establishing centers of excellence in genomics that provide research, training, and clinical care services could also significantly accelerate this goal. Partnerships between governments, academic institutions, private biotechnology companies, and international organizations will also be key. The South African Medical Research Council (SAMRC) genomics platform, with its dual arms of health service delivery and biomedical research, is a good example of the proposed model. Similar efforts to connect research with healthcare delivery have also shown promising outcomes. For example, in Cameroon, a genetics research program for SCD was used to pilot prenatal genetic testing for sickle cell disease [41] while in Ghana, research on the genetics of hearing impairment has informed policy recommendations for integrating rapid, cost-effective molecular diagnostics into the country’s universal newborn hearing screening program [42].

## Academic-Industry Genomic initiatives in Africa

Given the resource-intensive nature of genomics research and the limited funding for science and innovation in Africa, academic institutions alone cannot advance the genomic medicine agenda. Globally, academic-industry partnerships are becoming increasingly becoming prevalent [43–46]. In much of Africa, these partnerships often focus on transactional agreements, such as contracts for the commercial supplies of reagents, testing kits, data storage facilities and sequencing technologies. They rarely extend to supporting the translation of research findings into practical applications. It is time to move beyond these transactional models towards academic-industry partnerships where industry stakeholders actively fund activities like training, capacity building and translational genomics research in Africa.

Commercial biotech companies are becoming key players in the African genomics landscape. For instance, Variant Bio has partnered with the Africa Wits-INDEPTH partnership to establish an Expression Quantitative Trait Loci (eQTL) database based on South African genomic data [46]. Similarly, the commercial genomic startup, 54Gene has contributed to the Nigerian 100K genomes project, while Illumina Inc. collaborates with African scientists to expand access to whole genome sequencing for research initiatives [7, 45]; while Illumina Inc. is collaborating with a number of African scientists and genomic initiatives to expand access to whole genome sequencing for research projects [47, 48]. Beyond these partnerships, some companies also generate genetic data through the provision of clinical and research services. Despite these contributions, there is an urgent need to transition towards more equitable academic-industry partnerships in Africa. Such partnerships should be governed by frameworks that 1) enable academic researchers and other stakeholders to access genetic data generated by commercial entities; 2) clearly define the ethical obligations for companies regarding funding for research training, data ownership, benefit sharing, and intellectual property rights; 3) strike a balance between the objectives of academia, which focus on advancing scientific knowledge and societal benefits, and the commercial interests of industry partners.

## Commercialization of Genomics Knowledge

Genomics companies and biotech startups in Africa, such as BixBio (https://www.bixbio.com/) and Syndicate Bio (https://www.syndicate.bio/about-us/) are emerging as key contributors to genomics-enabled research and development. Despite this progress, the translation of genomics solutions from research settings to the marketplace and clinical environments remains notably slow. Addressing the factors behind this sluggish pace should be a pressing priority for realising the vision of “Genomics for All.” This requires a thorough evaluation of current practices and incentives for commercializing academic research within African universities and research institutions.

Discussions around commercialization in African genomics have often been framed by concerns about benefit sharing, mainly equitable access to interventions by for study communities and whether African researchers and institutions will directly benefit from the commercialization of genomic innovations. A recent controversy involving the development of a potentially commercialisable intervention using African samples and genomic data [49, 50], is a use case and exemplifies the need to address ethical and integrity issues in academic-industry partnerships in genomics research. It also reinforces the importance of carefully considering ethical and legal issues in African research initiatives that seek to commercialize the outcomes of genomics research. The H3Africa’s typology of commercialization in genomics [51] provides guidance for balancing the drive for innovation in genomics research with ethical considerations to ensure that commercialization efforts prioritize fairness, benefit-sharing, and the consent of study communities or participants.

## International Cooperations for the Promotion of Genomic Medicine in Africa

The implementation of genomic medicine in Africa, with its significant resource demands, calls for robust international cooperation and partnerships. Collaboration between African nations and global stakeholders can offer the financial, technical, and infrastructural support needed to scale up genomic medicine initiatives (Table 1). A prime example is the framework for implementing genomic medicine for public health in Africa [52], developed by the African Union Development Agency-NEPAD (AUDA-NEPAD) and the African Academy of Sciences, with financial support from the Bill and Melinda Gates Foundation. This framework emphasizes the need for a coordinated, continent-wide approach to integrate genomic medicine into public health systems, focusing on capacity building, infrastructure development, and equitable access to genomic technologies. Recent collaborations have also highlighted the role of science diplomacy in advancing genomic medicine. For instance, partnerships like the one between H3Africa and major global funders, such as the NIH and the Wellcome Trust, have fostered the creation of genomic research infrastructure across Africa, catalyzed the development of local expertise and research programs and supported the development of ethical governance frameworks to ensure fair benefit sharing and responsible data use. Also, bilateral agreements between African countries and international organizations have proven effective. For example, the partnership between France's Ministry for Europe and Foreign Affairs and North African countries through the PerMediNA initiative showcases how academic and governmental collaborations can drive region-specific genomic advancements. While partnerships like the one between AUDA-NEPAD and the EU-Africa Personalised Medicine project, further epitomises the value of bilateral science collaborations in advancing precision medicine in Africa.

**Table 1:** Summary of pathways to equitable access and delivery of genomic medicine in Africa

Pathway	Description	Key Strategies
The Diversity Genomic Data Imperative	Ensure African populations are represented in global genetic datasets	• Establish initiatives that target genomic studies among underrepresented populations • Strengthen collaboration between African genomic projects to improve data collection and sharing
Minimize Hoarding of Genetic Data	Equitable data sharing across all genomic research stakeholders	• Promote discussions on ethical obligations in sharing commercially or academically generated genetic research data • Develop frameworks and agreements for responsible data sharing across national borders • Incentivize data-sharing and encourage the use of platforms that allow for local stewardship and control of data while facilitating data analysis
Translational Genomics Research	Applying genomic discoveries and research outcomes directly into clinical practice and public health initiatives	• Support genomic research that combines short-term clinical utility with long-term impact • Fund programs that translate existing genetic knowledge into accessible, cost-effective healthcare solutions tailored to local populations
Build a Genomic Medicine Workforce	To develop a multidisciplinary workforce to support genetic medicine delivery	• Revise medical curricula to integrate genetics education • Expand training programs for genetic medicine in Africa • Continuous education genetics and ethical issues for healthcare providers
Academic-Industry Partnerships	Collaborations between academia and industry	• Promote equitable partnerships that for share resources, data, and expertise • Develop policies for addressing ethical issues in academic-industry partnerships • Industry partners should commit funding and support for academic research and training in genomic medicine in Africa
Commercialization of Genomics Knowledge	Translation of research into practical applications in healthcare	• Evaluate practices and incentives for commercialization in African universities • Develop a typology for responsible translation and commercialization of the outcome of genomics research
Public Engagement and Education	Engage communities and improve public literacy and understanding of genomics	• Develop genomic literacy programs for the lay public and patient support groups • Incorporate indigenous knowledge on genetics into research design and implementation
Science Diplomacy	Leverage global health leadership to advocate for genomic medicine implementation in Africa	• Build bilateral and multilateral partnerships to support genomic medicine implementation in Africa • Establish international treaties and agreements for data sharing and access to genomic innovations • Establish sustainable genomic funding mechanisms through partnerships with governments, international organizations, and private sector funders

## Conclusion

Science diplomacy and equitable academic-industry partnerships will be instrumental in ensuring that genomic solutions for medicine and healthcare are accessible to populations across Africa. The WHO Science Council has commmited to use its leadership role in global public health to advocate for broader availability of genomics medicine in member states [39]. However, achieving this vision requires unified and sustained efforts from African governments, academic institutions, funding agencies, philanthropists, commercial biotechnology companies, civil society, and international health organizations. These stakeholders must collaboratively define and implement a sustainable pathway to support genetic research in Africa; enhance the availability of genetic data from African populations; and promote translational genomics medicine initiatives tailored to the continent’s healthcare priorities. Strong leadership, inclusive of diverse voices from the scientific community, will be essential to advance discussions around “Genomics for All” and science advocacy efforts must prioritise the development of mechanisms to prevent the hoarding of genetic resources, including genetic data and new interventions, and ensure that the scientific, medical, and economic benefits of genomics are equitably distributed among all nations. Drawing lessons from the COVID-19 pandemic, African nations and the global community must work together to ensure that genomic resources and innovations are used responsibly, equitably, and for the collective benefit of humanity. This calls for international negotiations and treaties to govern access to genomic innovations; frameworks for responsible cross-border sharing of genomic data; equitable academic-industry partnerships; and long-term strategies to address funding gaps for both the conduct and translation of genomics research in Africa.

## Data Availability

No datasets were generated or analysed during the current study.
